# Telemedicine in Palliative Care: Implementation of New Technologies to Overcome Structural Challenges in the Care of Neurological Patients

**DOI:** 10.3389/fneur.2019.00510

**Published:** 2019-05-24

**Authors:** Christiane Eva Weck, Katharina Maria Lex, Stefan Lorenzl

**Affiliations:** ^1^Krankenhaus Agatharied GmbH, Hausham, Germany; ^2^Institut für Pflegewissenschaft und -Praxis, Paracelsus Medizinische Privatuniversität, Salzburg, Austria; ^3^Palliative Care, Ludwig Maximilian University of Munich, Munich, Germany

**Keywords:** neurological, telemedicine, neuropalliative care, specialized outpatient palliative care team, videoconsultation

## Abstract

Telemedicine provides a possibility to deal with the scarcity of resources and money in the health care system. Palliative care has been suggested to be appropriate for an increasing number of patients with neurodegenerative disorders, but these patients often lack care from either palliative care or neurology. Since palliative care means a multidisciplinary approach it is meaningful to use palliative care structures as a basis. There exists no systematic access to neurological expertise in an outpatient setting. A successful link of two existing resources is shown in this project connecting the Department of Neurology of an University Hospital with specialized outpatient palliative care (SPC) teams. A videocounselling system is used to provide expert care for neurological outpatients in a palliative setting.

**Methods:** A prospective explorative single arm pilot trial was implemented to provide a mobile telesystem for 5 SPC teams. The opportunity was given to consult an expert in neuropalliative care at the specialized center in the hospital (24/7). Semistructured interviews were conducted with the physicians of the SPC teams after a trial duration of 9 months.

**Results:** Our data provides strong evidence that the technical structure applied in this project allows a reasonable neurological examination at distance. Qualitative interviews indicate a major impact on the quality of work for the SPC teams and on the quality of care for neurological patients.

**Conclusion:** The system proves to be useful and is well accepted by the SPC teams. It supplies a structure that can be transported to other disciplines.

## Background

A multidisciplinary palliative care approach improves patient's quality of life and symptoms in advanced neurological diseases (1). End of life care in neurological diseases is often challenging since disease trajectories are less predictable compared to cancer patients (2). Furthermore, either the palliative care expertise might be lacking in neurologists or the neurological expertise be missing in palliative care experts (3).

Outpatient Palliative Care services are a multidisciplinary approach with a network around the core team. A specialized outpatient palliative care (SPC) support which enables patients to stay at home is currently seen as the most appropriate form of palliative care. Usually, the neurological expertise is lacking in SPC teams which makes it difficult to handle patients with either neurological diseases or neurological symptoms. In most countries no regulated approach to a neurological consultant in an outpatient setting is established.

Owing to the increased awareness of the benefits of palliative care for non-cancer patients, telemedicine might provide a solution to cope with the growing requirements in the health care system. It enables the provision of expert medical opinion over long distances and can transport the support to virtually any place. It offers the opportunity to enhance quality and capacity of medical care (4). Especially in rural areas a lack of experts due to a lack of human resources could be overcome by providing expertise via telemedicine. It gives the possibility to monitor patients with advanced illnesses at home (5).

Here we describe an established system that provides the opportunity to consult an expert in neurology/neuropalliative care via a teleconference app.

## Methods

### Study Design

A single center, multi-site, non-randomized trial was conducted at a Bavarian Neurological Medical Centre with expertise in neuropalliative care. Five teams were equipped with a mobile telesystem to consult an expert in neuropalliative care at the specialized center (24/7). The mobile telesystem allows a videoconsultation between the patient at home and the medical center. Patients had to meet the following criteria: (1) to be attended by one of the five selected specialized outpatient palliative care teams with a (2) diagnosis of a neurological disease or having a cancer diagnosis suffering from neurological symptoms. Ethics were approved by University Ethics committee (Nr. 17-068) and the study was registered at the DRKS.

**Figure 1:**
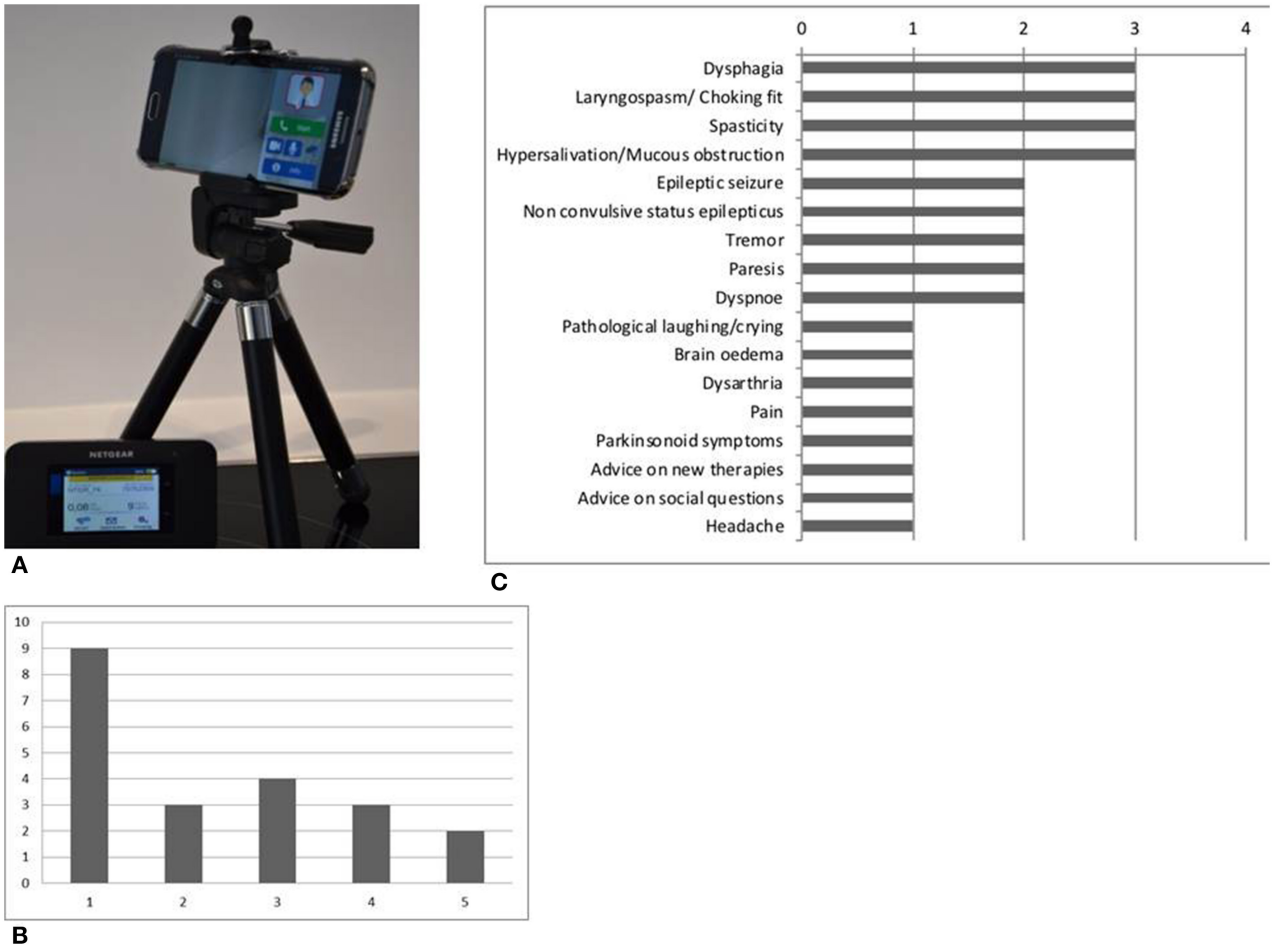
**(A)** Teleconsultation device consisting of a mobile phone, a small tripod and a mobile WIFI router; **(B)** Number of patients co-supervised per SPC team (team 1 to 5 on the x axis); **(C)** Frequence of symptoms discussed in the consultations.

Each team selected has been equipped with a mobile teleconsultation device consisting of a mobile phone with a high resolution camera (Samsung Galaxy S6) supplemented by a mobile WIFI router and a small tripod (see Figure 1A). Additionally, a WIFI router which offers the opportunity to generate a wireless LAN, has been supplied.

The videoconference software (MEYDOC®) is installed on the mobile device as an app. The acquired software ensures high data integrity providing a point to point communication with authenticated endpoints, the server is used only for call control procedure and an end to end encryption is used. The teleconsultation equipment at the medical center consists of a laptop with the videoconference software installed.

### Intervention

Teleconsultations are held on demand. When the outpatient team identifies a symptom which is difficult to control, a call is made to the expert medical center for an appointment.

Depending on the acuteness of the problem the teleconsultation usually is scheduled within the next 1 to 5 days. Generally, the teleconsultations can be scheduled beforehand. However, emergency calls are possible (24 h/day). The screen to screen contact is built up between the patient's home and the specialist in the medical center. The hospital-based neurological team consists of two neurologists (one having major expertise in neuropalliative care). A physician or a nurse of the specialized palliative outpatient team is involved by joining the video consultations at the patient‘s home.

For data analysis we have used a mixed methods approach. Quantitative data: We have documented personal data of the patients, the neurological diagnosis, main neurological symptoms, and the technical quality of the teleconsultation (ranking by the physicians at the medical center on a NRS 1-5). For qualitative analysis we have used a semistructured interview guide (see Table 1a).

**Table 1a:** Interview guide.

**Questions**
1) How did you handle neurological problems prior the participation in the trial?
2) Did the project influence your job satisfaction?
3) Did the project modify your daily job activity? If yes, how did it change your work?
4) How do you estimate your knowledge concerning neurological problems prior to and post-trial participation?
5) Did you have problems with the technology, which were the most disturbing ones?
6) Did you think there was a problem with the patient‘s acceptance of the telemedical system?
7) Do you have any suggestions for the next trial stage?
8) Miscellaneous

After a trial duration of 9 months the researchers conducted five semi-structured ethnographic interviews with the leading physician of each specialized palliative care team. The interviews were recorded, transcribed and anonymized. They were subjected to a pragmatic thematic analysis of the content conducted by CW and KL.

## Results

### Specialized Outpatient Palliative Care Teams–Selection and Characterization

We asked seven SPC teams in Bavaria to participate. Finally, five teams agreed to participate (One team never answered the proposal. The other team already had the support of a neurologist.) The teams covered an area of about 7,250 km^2^ (ranging from 317 to 2,370 km^2^) with a population density ranging from 113 inhabitants per km^2^ to 4,713 inhabitants per km^2^, employing from two to 5.4 physicians. In the five participating teams the specializations consist of anaesthisiologists (9), general practitioners (6), internists (7), and one geriatrician. In one team a neurologist stepped in during the ongoing trial.

### Technical Feasibility

The first 26 videoconsultations were evaluated for their technical quality. A stable connection with a satisfactory quality of the visual and acoustic components even in rural areas is feasible using the dual phone card solution and the mobile wireless LAN router (NRS 2). In two cases problems occurred with the audio line. In some cases the pre-existing wireless LAN of the patient‘s home was utilized. Redialling was sometimes necessary to establish the connection. However, in every case a teleneurological consultation with sufficient quality to determine the acute problems and to make a neurological assessment was possible.

### Quantitative Data

Until March 2018, 37 teleconsultations were held concerning 21 patients. Eleven of the consultations were conducted via telephone, 26 consultations via videoconference. Figure 1B shows the number of patients co-supervised per team varying from nine to two patients. In 48% of the cases a re-consultation was conducted with up to 4 follow up consultations for one patient. Fourteen of the 21 patients were cared for by the SPC for neurological disorders. The other seven patients had an internal or oncological diagnosis and neurological symptoms. Half of the patients suffered from motor neuron disease, three of them from glioblastoma. The other four had Parkinson‘s disease, Progressive supranuclear palsy, a non-convulsive status epilepticus and unclassified dementia. Figure 1C shows the main neurological symptoms discussed in the video and telephone consultations from all of the 21 patients. The leading symptoms were dysphagia, hypersalivation, laryngospasm, spasticity, and epileptic seizures or non-convulsive status epilepticus.

### Qualitative Data

A positive impact of the telemedical project for the teams and the patients is the core tenor of the interviews. The SPC teams perceived that the patients highly accepted a neurological telemedical visit. Recommended therapy procedures, discussed in the teleconsultation often led to efficient symptom control thereby improving patient's quality of life, as perceived by the SPC teams.

Even if there has been no improvement with the suggested treatment, the fact that everything possible was done by consulting a specialist, has been significant enough to have a positive effect on the patient‘s satisfaction. Physicians experience an obvious increase in the satisfaction with the quality of their work. SPC teams feel safer having a neurological telemedical background. There was no clear structure in handling neurological/neuropalliative questions in any of the teams prior to the participation in this trial. Strategies used before the telemedical application included asking the residential neurologists or the nearest neurological department, reading books and making treatment decisions on their own. Therefore, using the telemedical application even these structures for the teams have been improved. The project changed the awareness of neurological symptoms, it resulted in a faster consultation. It has been highly acknowledged to have a contact person with neuropalliative care expertise. To further point out: a key feature of the telemedical approach with a huge significance is the visual component of the consultation.

Suggestions for technical improvement were a bigger display for the videosystem at the patient's side and the request for a timely fixed consultation hour beyond the videoconsultations, for short discussions concerning neurological symptoms or medications (quotes of the interviews are listed in Table 1b).

**Table 1b:** Quotes of the semistructured interviews.

Patient‘s acceptance of the neurological telemedical screen to screen visit	“…if we inform patients that we want to consult a neurologist, who is unable to come in person but joins us via a videoconference, patients are actually enthusiastic”(interview C, line 57–58)
	“…they rather thought this was a really good idea and were excited, because when suffering from ALS or MS they no longer manage to visit the resident neurologist.”(interview D, line 192–194)
Symptom control by recommended therapy	“Patient X… she lived quite a long time with a significant increase in mobility and was very satisfied and extremely thankful.” (interview C, line 15)
The fact that everything possible was done by consulting a specialist	“patients are highly satisfied also because they feel comprehensively cared for.” (interview A, line 41)
Satisfaction with the quality of their work increases in the SPC teams	“where a new neurological symptom supervenes … and I feel incapable of making the right diagnosis and initiating the accurate therapy … it is really brilliant for this.”(interview C, line 34–37)
	“in other cases there were fewer consequences (*therapeutically*), but we got certainty”(interview C, line 11–12)
Clear structures make it easier to discuss neurological problems	“if we have a reasonable initial suspicion“ (interview C, line 23–24).
	“It was extremely helpful, we may never have solved such questions” (interview D, line 125–126).
The visual component is a key feature of the system	“… asking you without inhibitions, and not only calling and describing, but really displaying, having you with us in the living room (*via camera*)” (interview D, line 26–27).

## Discussion

In this small pilot study we have been able to show for the first time that telemedical support for SPC teams with a focus on neurological patients or neurological symptoms in oncology patients is technically feasible and supports the team's treatment. It enables the teams to get rapid access to neurological and neuropalliative care expertise without losing contact to the patient. Until now, there was no clear structure in the teams in dealing with these issues which often caused troubles since neurological expertise is usually only available during hospital treatment. However, since patients with progressed neurological diseases are usually bedridden and have severe communication problems they are frequently difficult to transport to a hospital or even a palliative care unit. Telemedical consultation therefore enabled the patient to stay at home and the SPC team to be the primary provider of care using expert opinion on demand. This also strengthened the relationship between the patient and the SPC team.

Patients with a neurological diagnosis are seldom cared for by SPC teams. Due to the growing awareness of the usefulness of a multidisciplinary palliative approach in progressive neurological conditions, we suggest a growing number of neurological patients in the specialized outpatient teams. The telemedical project offered clearly defined consultance structures which also improved the quality of work and job satisfaction of the SPC teams. The interviews with the physicians report a high acceptance of the telemedical application by the patients. It is important to point out that in some cases where we couldn't add much to symptom control, only the patient's awareness of comprehensive medical care brought benefit to the patient. To get an unbiased view of patient's acceptance further interviews with the patients and caregivers have to be performed. The offered system, especially because of the possibility of a visual way of appraisal, yields more safety in the care for neurological palliative outpatients. Furthermore, the system is small, easy to carry and it stands out due to a simple application.

Since this is a pilot trial, the number of patients is too small for statistical analysis. Not surprising is the fact that half of the patients with a neurological diagnosis cared for by SPC teams suffer from ALS. This is one of the few neurological diagnoses where the need and the benefit of a palliative support is already comprehensively proven (7). Therefore, the main symptoms discussed in the videoconsultations were pseudohypersalivation, laryngospasm/choking fits, dyspnoea, and spasticity.

The concentration on neurological and neuropalliative care questions and the encompassed needs in a palliative situation proves successful. A comprehensive palliative care approach can be difficult to provide via telemedicine as shown in a telemedical approach for pediatric palliative care (6). Further application might provide access to specialist in cardiac or pulmonary care (8).

The system we offer works even in rural area. The technical construction (two mobile cards and the WIFI router) is stable enough even with a low bandwidth. As a future task, we are currently preparing to include more SPC teams as we have seen that based on the amount for videoconsultation we can provide our knowledge to an even larger number of teams. The suggested improvements (bigger display of the videotool and a consultation hour) will be implemented.

In conclusion, the qualitative interviews suggest that expert neurological and neuropalliative consultation is helpful in SPC teams concerning patients quality of life and the quality of work for the SPC teams. Our telemedical approach offers technical components which are easy to handle and have stable communication lines even in remote areas. The telemedical “home visitation” of a specialized neurologist has been well accepted by the teams. It provides an easy and effective way of symptom discussion and treatment evaluation. Further research is needed to explore telemedical applications in palliative care consultations.

## Ethics Statement

Ethics committee Ludwig Maximilians University Munich. 17-068, 6.6.17. Written informed consent was obtained from all participants in this study.

## Author Contributions

CW and SL are the Researchers in the Project. The Project was initiated by SL and CW was part of the Project from the beginning. KL and CW evaluated the qualitative Research part.

### Conflict of Interest Statement

The authors declare that the research was conducted in the absence of any commercial or financial relationships that could be construed as a potential conflict of interest.
